# Synovitis in osteoarthritis: current understanding with therapeutic implications

**DOI:** 10.1186/s13075-017-1229-9

**Published:** 2017-02-02

**Authors:** Alexander Mathiessen, Philip G. Conaghan

**Affiliations:** 1 Department of Rheumatology, Diakonhjemmet Hospital, Oslo, Norway; 2 Leeds Institute of Rheumatic and Musculoskeletal Medicine, University of Leeds, Leeds, UK; 3 National Institute for Health Research Leeds Musculoskeletal Biomedical Research Unit, Leeds, UK

**Keywords:** Osteoarthritis, Synovitis, Pathophysiology, Epidemiology, Imaging, Treatment

## Abstract

Modern concepts of osteoarthritis (OA) have been forever changed by modern imaging phenotypes demonstrating complex and multi-tissue pathologies involving cartilage, subchondral bone and (increasingly recognized) inflammation of the synovium. The synovium may show significant changes, even before visible cartilage degeneration has occurred, with infiltration of mononuclear cells, thickening of the synovial lining layer and production of inflammatory cytokines. The combination of sensitive imaging modalities and tissue examination has confirmed a high prevalence of synovial inflammation in all stages of OA, with a number of studies demonstrating that synovitis is related to pain, poor function and may even be an independent driver of radiographic OA onset and structural progression. Treating key aspects of synovial inflammation therefore holds great promise for analgesia and also for structure modification. This article will review current knowledge on the prevalence of synovitis in OA and its role in symptoms and structural progression, and explore lessons learnt from targeting synovitis therapeutically.

## Background

Osteoarthritis (OA) is the most common form of arthritis and a major cause of joint pain and disability. We live longer than our ancestors and, for the first time in history, people aged 65 years and older will outnumber children younger than 5 years, and the number of people aged 60 years and above is expected to double by 2050 and more than triple by 2100 [1]. Being primarily related to ageing, the prevalence of OA will steadily increase and is expected to be the single greatest cause of disability in the general population by 2030 [2]. This will not only affect individuals’ quality of life but also account for substantial burden on health care systems globally. Yet current analgesic therapies are limited in efficacy and by significant toxicity and there are no licensed disease-modifying drugs.

In the early 1980s, histopathological analysis of OA synovium demonstrated abundant inflammation in the majority of OA patients [3]. While traditionally considered primarily a disease of hyaline cartilage with associated bone involvement, caused by overload or overuse, the pathophysiology of OA development is now appreciated to be more complex. Mounting evidence suggests that synovitis and the resultant pro-inflammatory mediators are important in the pathogenesis of OA with effects on articular cartilage [4, 5]. Modern imaging modalities such as magnetic resonance imaging (MRI) and ultrasound have confirmed a high prevalence of ‘macroscopic’ inflammation and have supported the role of synovitis as an active component of the OA process, associated with both pain and structural progression (Table 1).

**Table 1:** Evidence for the role of synovitis

Level of evidence	Observation	References
Clinical	Effusion, joint swelling or palpable synovitis	Thomas et al. [2], Sellam et al. [5], de Lange-Brokaar [39]
	Sudden increase in pain	
	Night pain and morning stiffness	
Histological	Synovial hypertrophy and hyperplasia	Goldenberg et al. [3], Prieto-Potin et al. [8], Klein-Wieringa et al. [9], Deligne et al. [10], de Lange-Brokaar et al. [11], Goldring [13]
	Infiltration of mononuclear cells (monocytes/macrophages, activated B cells and T cells)	
	Adaptive immune T-cell and B-cell responses to fragments of extracellular matrix	
	Macrophages cluster and form multinucleated giant cells for improved phagocytosis	
	Increased angiogenesis	
	Synovitis close to degenerative cartilage	
Molecular	Production and/or release of pro-inflammatory cytokines (TNF, IL-1β, IL-6, IL-8, IL-15, IL-17, IL-18, IL-21)	Sokolove and Lepus [12], Wojdasiewicz et al. [16], Larsson et al. [61], Pustjens et al. [18]
	Increased production of PGE2 and nitric oxide	
	Increased expression of adhesion molecules (ICAM-1, VCAM-1) in the synovium	
	Increased activity of MMPs (MMP-1, MMP-3, MMP-9, MMP-13) and ADAMTS	
	Production of adipokines (visfatin, leptin, adiponectin)	
	Release of EGF and VEGF	
	Involvement of macrophages in osteophyte formation via BMPs	
	Insufficient release of anti-inflammatory cytokines (IL-4, IL-10, IL-13, IL-1Ra)	
	Release of pro-inflammatory and pain neurotransmitters (substance P, NGF)	
Imaging	Gadolinium-enhanced synovium and increased synovial volume detected by MRI	de Lange-Brokaar et al. [21], Loeuille et al. [22], Sarmanova et al. [27], Mathiessen et al. [29], Haugen et al. [26], Kortekaas et al. [30], Yusuf et al. [35], de Lange-Brokaar et al. [39], Felson et al. [41], Damman et al. [49]
	MRI correlates with histological observations and joint volume by arthrocentesis	
	High prevalence of synovial hypertrophy and effusion using ultrasound	
	Association between MRI-detected and ultrasound-detected synovitis and clinical symptoms of synovitis	
	MRI-detected and ultrasound-detected synovitis predicts incident radiographic OA, progression and cartilage degradation	
Interventions	High dose of IA corticosteroid injection may have short-term effects on clinical symptoms and synovial tissue volume	Zhang et al. [53], O’Neill et al. [50], Keen et al. [56], Wenham et al. [58], Wenham et al. [59]
	Methotrexate may have an analgesic effect	
	Biological response modifiers have potentially structural-modifying effects	

Adapted from [5] with permission from Macmillan Publishers Ltd

*ADAMTS* a disintegrin and metalloproteinase with thrombospondin motifs, *BMP* bone morphogenetic protein, *EGF* endothelial growth factor, *IA* intraarticular, *ICAM-1* intercellular adhesion molecule 1, *IL* interleukin, *IL-1Ra* interleukin 1 receptor antagonist, *MMP* matrix metalloproteinase, *MRI* magnetic resonance imaging, *NGF* nerve growth factor, *OA* osteoarthritis, *PGE2* prostaglandin E2, *TNF* tumour necrosis factor, *VCAM-1* vascular cell adhesion molecule 1, *VEGF* vascular endothelial growth factor

In this narrative review, we aim to summarize current knowledge on the role of synovitis in OA with emphasis on recent research on pathophysiology and epidemiology, and the lessons learned from recent trials targeting synovial inflammatory mediators. We selected papers in English with relevance to peripheral joint OA and attempted to include updates on a variety of OA anatomical sites, although much of the literature focus remains on knees. Although we have focused on synovitis, it is important to note that pro-inflammatory mediators may also arise from multiple OA joint structures including the infrapatellar fat pad.

## Synovial pathophysiology

### Normal synovium

The synovium is a specialized connective tissue that lines diarthrodial joints, surrounds tendons and forms the lining of bursae and fat pads. In synovial joints, the synovium seals the synovial cavity and fluid from surrounding tissues. The synovium is responsible for the maintenance of synovial fluid volume and composition, mainly by producing lubricin and hyaluronic acid. Through the synovial fluid, the synovium also aids in chondrocyte nutrition (together with subchondral bone), as articular cartilage has no intrinsic vascular or lymphatic supply [6].

The normal synovium has two layers. The outer layer, or subintima, is up to 5 mm thick and consists of multiple types of connective tissues: fibrous (dense collagenous type), adipose (found mainly in fat pads) or areolar (loose collagenous type). This layer is rich in type I collagen and microvascular blood supply, accompanied by lymphatic vessels and nerve fibres, but is relatively acellular [7]. The inner layer, or intima, lies next to the joint cavity and consists of a layer of 1–4 cells, only 20–40 μm thick. These synoviocytes have been identified by immunohistochemical and cytochemical methods as macrophages and fibroblasts; the latter is the dominant cell population in healthy synovium [7].

### Changes in OA synovium

The histological pattern of synovium in OA patients is characterized by synovial lining hyperplasia, sublining fibrosis and stromal vascularization [8]. There is an abundant influx of leukocytes from the vascular compartment in response to cytokines and cell adhesion molecules [6], and several studies have shown macrophages and T-cell lymphocytes to be the most predominant immune cells in OA synovium, whereas mast cells, B cells and plasma cells are also found but to a lesser extent [9–11].

Macrophage infiltration in the synovium is common in both OA and RA [8]. These macrophages can cluster and form multinucleated giant cells (MGCs) for improved phagocytosis, and are increased in similar numbers in inflamed OA and RA synovia compared with non-inflamed OA and post-mortem controls [8]. However, OA and RA demonstrate slightly different subgroups of MGCs, suggesting different drivers for these clusters, such as more cartilage debris in OA.

**Fig. 1:**
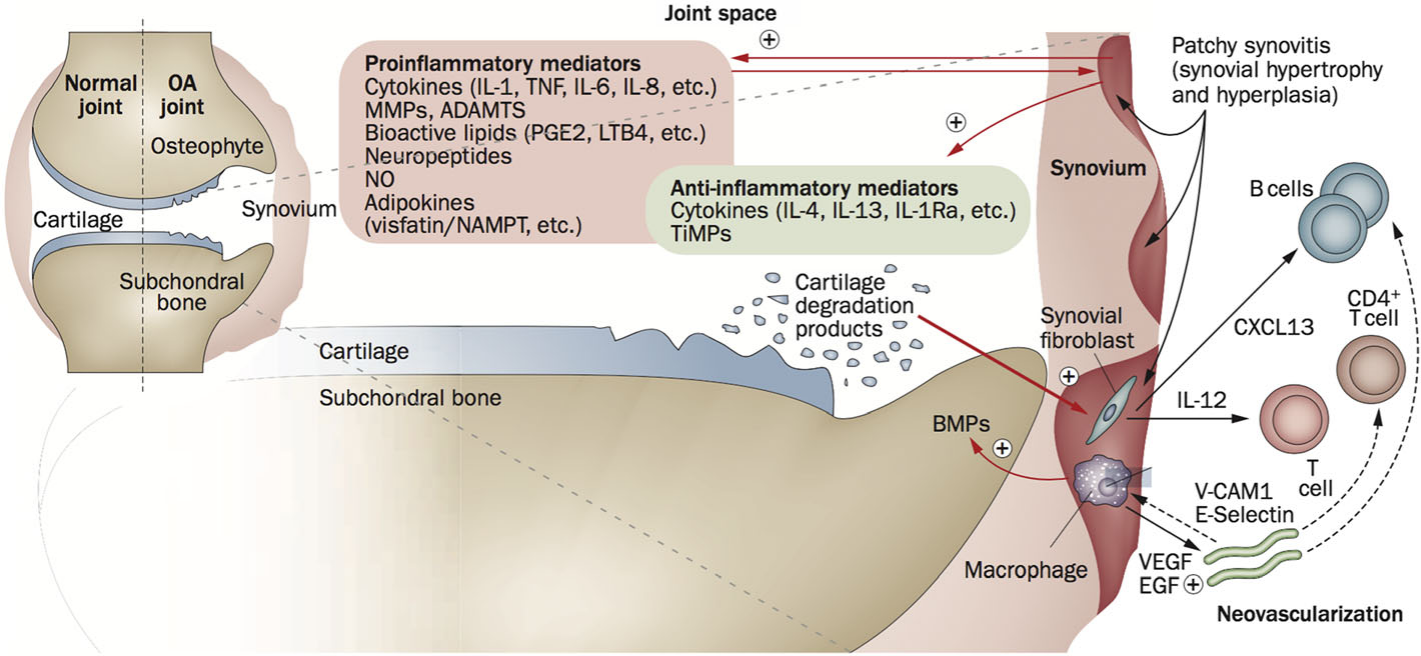
Involvement of the synovium in OA pathophysiology. Products of cartilage breakdown released into the synovial fluid are phagocytosed by synovial cells, amplifying synovial inflammation. In turn, activated synovial cells in the inflamed synovium produce catabolic and pro-inflammatory mediators that lead to excess production of the proteolytic enzymes responsible for cartilage breakdown, creating a positive feedback loop. The inflammatory response is amplified by activated synovial T cells, B cells and infiltrating macrophages. To counteract this inflammatory response, the synovium and cartilage may produce anti-inflammatory cytokines. In addition to these effects on cartilage inflammation and breakdown, the inflamed synovium contributes to the formation of osteophytes via BMPs. *ADAMTS* a disintegrin and metalloproteinase with thrombospondin motifs, *BMP* bone morphogenetic protein, *CXCL13* CXC-chemokine ligand 13, *EGF* endothelial growth factor, *IL* interleukin, *IL-1Ra* IL-1 receptor antagonist, *LTB4*, leukotriene B4, *MMP* matrix metalloproteinase, *NAMPT* nicotinamide phosphoribosyl transferase (visfatin), *NO* nitric oxide, *OA* osteoarthritis, *PGE2* prostaglandin E2, *TiMP* tissue inhibitor of metalloproteinase, *TNF* tumour necrosis factor, *VCAM-1*, vascular cell adhesion molecule 1, *VEGF* vascular endothelial growth factor (Reprinted from [5] with permission from Macmillan Publishers Ltd)

Much of the innate immune activation and cytokine production in the OA joint is attributed to synovial macrophages, but other cells including synoviocytes and chondrocytes also play a role [12]. The underlying mechanisms are complex and beyond the scope of this review. In short, molecules from degraded hyaline cartilage released into the synovial cavity are likely to initiate synovial inflammation in OA (Fig. 1). Early in knee OA, damage to the meniscus may also release tissue debris, although molecules released from subchondral bone may also play a role. Synoviocytes react by producing pro-inflammatory mediators, which in turn attract immune cells, increase angiogenesis and induce a phenotypic shift in chondrocytes [13]. A vicious cycle follows, as chondrocytes produce additional cytokines and proteolytic enzymes that eventually increase cartilage degradation and induce further synovial inflammation [4].

The processes driving inflammation in OA are complex. Given that OA is age related, immunosenescence may play a role in the immune response to tissue damage. A recent report analysed immune cell composition of the blood of OA patients and found compromised immune function of T cells and B cells beyond what appeared directly related to ageing, and this could reflect both inflammation and autoreactivity [14]. Also, trauma can trigger release of local inflammatory mediators, and there is increasing evidence that metabolic syndrome and obesity increase systemic low-grade inflammatory mediators which may synergize with other inflammatory mechanisms in OA [15].

### The role of cytokines

Wojdasiewicz et al. [16] recently described in detail the mediating cytokines and their signalling pathways that are up-regulated in OA and most often have catabolic (i.e. degrading) effects, including interleukin-1 beta (IL-1β), tumour necrosis factor (TNF) alpha, IL-6, IL-15, IL-17 and IL-18. IL-1β and TNF-α have been the most extensively studied cytokines. They are elevated in OA synovial fluid, synovial membrane, cartilage and subchondral bone and, in the course of OA, have synergistic effects on signalling pathways that increase inflammation and cartilage degradation [16]. Despite these known actions, anti-TNF treatment has not provided symptomatic benefits in OA randomized trials, although it has reduced structural progression in individual joints with high degrees of inflammation, whereas IL-1β inhibition has so far been disappointing in terms of symptom and structural benefits.

Another group of ‘haematopoietic’ cytokines, or colony-stimulating factors (CSFs), is known to activate and mature myeloid cells both systemically and locally [17], and there is an increased interest in targeting these CSFs in inflammatory and autoimmune diseases. During an inflammatory response, they act more distinctly and restricted on cell receptors than other cytokines, causing increased cell numbers of selected myeloid populations and enhancing their survival or modifying tissue destinations. Preliminary data show beneficial effects of anti-CSF therapies in OA models, and a phase II trial in inflammatory hand OA has been initiated [17].

Some cytokines (such as IL-4, IL-10 and IL-13) have anti-inflammatory and sometimes anabolic effects, and may modulate an inflammatory response and slow progression of OA [16]. In a healthy joint, the balance between anabolic and catabolic cytokines contributes to stable turnover of cartilage, whereas an imbalance is found in OA. Although the literature is limited, there is emerging evidence that an up-regulation of these cytokines, either individually or combined, may induce cartilage repair in OA. A novel fusion protein of IL-4 and IL-10 has induced both structural repair and reduced inflammation ex vivo, and inhibited pain in animal models of OA [18].

## Epidemiology

There is extensive evidence for synovitis being a common feature in OA. Tissue histology and immunohistochemistry have been used to identify synovitis and modern imaging techniques such as MRI and ultrasound are capable of visualizing aspects of synovitis, such as increased vascular flow, hypertrophic synovium and effusion. In addition, semi-quantitative scoring systems have enabled improved understanding of synovitis in OA pathogenesis and progression [19]. The prevalence of synovitis in OA joints depends on the diagnostic technique applied, on patient/cohort selection and on OA structural severity. However, prevalence studies do not often capture non-steroidal anti-inflammatory drug (NSAID) use, and it is possible that the anti-inflammatory action of these agents could lead to an underestimation of synovitis.

### Microscopic assessment of synovitis

OA synovial tissue typically displays a mild to moderate degree of inflammation on standard histological staining [20]. In smaller histological studies, the prevalence of inflamed synovium ranges greatly from approximately half to nearly all tissue samples depending on patient pre-selection and OA severity [11]. When comparing microscopic synovial changes in early and late OA, the literature has shown conflicting results [11], probably due to different definitions of ‘early’ and ‘late’ OA.

### Macroscopic assessment of synovitis

**Fig. 2:**
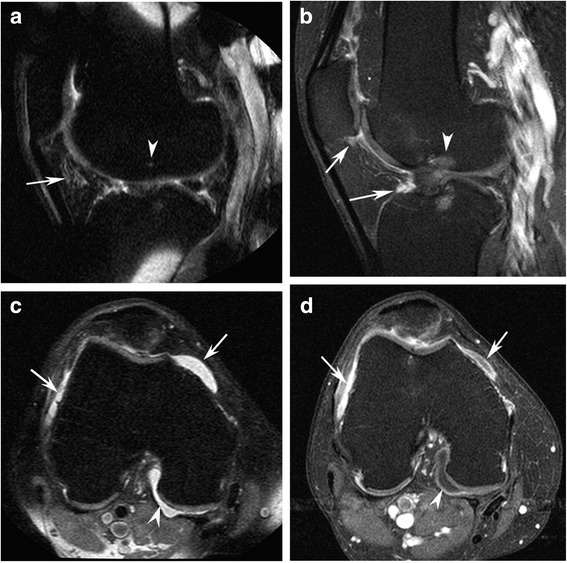
**a** Sagittal fat-suppressed proton density-weighted image (non-CE-MRI) shows hyperintensity in the intercondylar (*arrow*) region of Hoffa’s fat pad. This signal alteration is used as a surrogate marker for synovitis on non-CE-MRI. There is a discrete subchondral bone marrow alteration in the femur (*arrowhead*) corresponding to the site of the anterior cruciate ligament (ACL) insertion. **b** In contrast, sagittal fat-suppressed T1-weighted contrast-enhanced MRI (CE-MRI) shows no intercondylar synovitis, but reveals infrapatellar synovitis and synovitis adjacent to the tibial ACL insertion (*arrows*) not seen on non-CE-MRI, as well as bone marrow edema (*arrowhead*). **c** Axial non-CE-MRI shows a fluid-equivalent signal within the joint cavity suggestive of joint effusion in the peripatellar recesses (*arrows*) and posteriorly (*arrowhead*). **d** Axial CE-MRI shows marked synovitis anteriorly (*arrows*) and true effusion only depicted posteriorly as hypointensity adjacent to the synovial lining (*arrowhead*). Reprinted from [62] with permission from Elsevier

At the ‘macroscopic’ level, MRI provides valuable insights into synovitis and can visualize synovial hypertrophy, synovial fluid volume and level of synovial enhancement after intravenous injection of a contrast agent (Fig. 2). In terms of validity, MRI inflammation measures correlate well with histological inflammation and with effusion volume on arthrocentesis [21, 22]. In over a thousand knee OA patients, non-contrast-enhanced (non-CE)-MRI synovitis was present in 60% and effusion in 66% [23]. On CE-MRI, synovitis has been strongly correlated with radiographic OA severity and was reported in 74% of 404 patients having all grades of knee OA [24], and in 95% of 125 patients with mainly moderate to severe radiographic OA [25]. With respect to meniscal damage, only severe lesions have shown borderline association with synovitis [24]. MRI scans of hand OA patients have demonstrated synovitis in a median (interquartile range) 6 (4–7) interphalangeal joints of the dominant hand, with moderate to severe synovitis (grade 2–3) being infrequent [26].

Although ultrasound cannot assess cartilage deep in the joint or intra-bone lesions, it is well suited to assess inflammatory changes. A large meta-analysis of ultrasound-detected synovial changes found that people with knee OA or knee pain had high prevalence of effusion, synovial hypertrophy and power Doppler signals (ranging from 33 to 52% of the knees), and that these measures correlated well with histological findings [27]. A large multicentre study using ultrasound in 600 people with symptomatic knee OA demonstrated synovial inflammation or effusion in 46% of the population, despite using a strict definition of synovial hypertrophy [28]. In two large hand OA cohorts, nearly all patients (94% and 96%) had evidence of sonographic synovitis by grey scale in at least one finger joint [29, 30] and by power Doppler signals in 42% of the patients [29]. At the joint level, 28.7% of 1078 finger joints with Kellgren & Lawrence grade ≥ 2 demonstrated synovial effusion and/or hypertrophy by ultrasound [29].

### Is inflammatory OA a separate subtype?

With MRI and US studies demonstrating a high frequency of synovitis, it remains unclear whether inflammatory OA is a distinct subtype or just part of the spectrum of OA pathology severity. The group with more severe inflammation can be identified with imaging and serum biomarkers (such as the matrix metalloproteinase-dependent degradation of C-reactive protein (CRPM), connective tissue type I collagen turnover (C1M) and matrix metalloproteinase 3 (MMP-3)) [15]. To add further complexity, the expression of a given phenotype also depends on person-specific factors such as local muscle strength and obesity. A study followed two selected groups of knee OA patients for 4 years: one with metabolic syndrome and one lean group with frequent physical activity [31]. These groups differed significantly in the MRI expression of joint pathology, in which Hoffa synovitis was more prevalent in the active lean group, and prepatellar bursa signal, osteophyte scores and cartilage damage scores were higher and more prevalent in the metabolic syndrome group [31]. Inflammatory OA is a term often applied to a subset of hand OA with a greater degree of synovial inflammation and associated with radiographic erosive disease [32].

## Synovitis and symptoms

With different approaches employed in the detection and characterization of synovitis (such as histologic or imaging assessment), overall the literature provides consistent evidence of an association between synovial inflammation and OA symptoms, although the strengths of such associations vary and often studies do not examine all involved OA pathologies to explore which tissues are independently contributing to pain. Synovial inflammation increases the responsiveness of peripheral nociceptive neurons, leading to heightened pain sensitivity, and thereby contributes to increased pain experience [23]. Local immune cells and cytokines may modulate this altered pain perception, as demonstrated in knee OA patients when synovial CD4^+^ T cells were associated with VAS pain (adjusted for age, sex and BMI) [9]. No association was found for other immune cells. Furthermore, these CD4^+^ T cells secreted TNF-α and IL-6, which have been shown to directly affect sensory fibre function.

Before considering structure–pain relationships, it is worth considering the many differences between studies. Especially in large joints such as the knees, synovitis is found in a patchy distribution, and distinct patterns of synovitis may have different relationships to pain [33, 34]. Also, just as there are differences in how we detect synovitis, there are differences in how we measure ‘pain’. In some studies, associations may be with VAS global pain intensity scores, in other studies with summed WOMAC pain scores. We still need to understand more about individual symptoms and their relationship to particular structural pathologies. Importantly, pain is a complex experience in which changes may be attributed to several peripheral nociceptive factors other than inflammation, as well as central factors.

### Cross-sectional data

A systematic review from 2011 found an increased risk of MRI-detected synovitis and effusion in patients with symptomatic knee OA [35], but none of the included papers employed CE-MRI, without which effusion and synovitis cannot be readily differentiated. There are, however, emerging data for an even stronger association with pain severity when contrast enhancement is used [36].

Several cross-sectional studies on hand OA have shown similar associations between sonographic or MRI-detected synovitis and concurrent pain. A hand OA cohort applying ultrasound found inflammatory features (i.e. grey-scale synovial effusion or hypertrophy and power Doppler signals) to be significantly and dose-dependently associated with pain upon palpation and with the Australian/Canadian Osteoarthritis Hand Index (AUSCAN) pain and stiffness subscales and the Short Form (SF)-36 [30]. These results were replicated with MRI in another cohort [26], suggesting that synovial inflammation can cause pain also in hand OA.

### Longitudinal data

The effects of synovitis on longitudinal measurements of pain are more complex to measure and results are ambiguous. Most studies have been performed on knee OA cohorts with 2–5 years of follow-up, often assessing VAS pain, WOMAC, KOOS or ICOAP. Some studies find an improvement in pain when some (but not all) aspects of synovial inflammation diminish [37] and a worsening of pain with increased inflammation over time [38], whereas others do not replicate these associations [39]. In hand OA patients, MRI-detected synovitis has been found to predict incident joint tenderness but not AUSCAN pain [40].

## Synovitis and structural progression

### Incident OA

The effect of synovitis on OA is confounded by concurrent pathologies, raising an important question: does synovitis have an independent effect on OA progression? Felson et al. [41] recently examined the risk for incident radiographic knee OA after adjusting for structural pathology known to cause synovitis. The authors compared 239 cases and 731 control knees in the MOST cohort and found that cartilage lesions, meniscal damage, synovitis and BMLs were all risk factors for OA. Furthermore, when adjusting for confounding pathologies, synovitis remained associated with incident radiographic OA when the total synovitis score was 3 or higher on a 0–9 scale (OR 1.6, 95% CI 1.2–2.1). Another paper from the same cohort showed that knees without OA (i.e. having neither MRI-defined cartilage damage nor tibiofemoral radiographic OA) had a significant increased risk of cartilage loss whenever effusion synovitis was present (OR 2.7, 95% CI 1.4–5.1), independent of confounders for inflammation and cartilage loss [42]. These results suggest that synovitis effusion assessed on non-CE-MRI is an independent predictor of incident radiographic OA.

A series of recent papers from the OA Initiative (OAI) also support the importance of synovitis prior to incident radiographic OA. Atukorala et al. [43] performed nested case–control analyses on 133 knee joints that developed radiographic knee OA and an equal number of joints that did not develop knee OA. The authors found effusion synovitis and Hoffa synovitis 1 year prior to diagnosis to be significantly associated with subsequent OA development (OR 3.23, 95% CI 1.72–6.06 and OR 2.40, 1.43–4.04). A similar design was applied by Roemer et al. [44], who looked at repeated MRI scans up to 4 years prior to evident radiographic knee OA. They found that presence of Hoffa synovitis, effusion synovitis, medial BMLs and medial meniscal damage increased the risk of OA 2 years prior to incident radiographic OA, and that the number of features present increased the risk more than the presence of any single feature. More studies examining the incidence of the earliest detected MRI changes of OA will be required to understand more definitely the role of synovitis in the early stages of OA.

### Progression of OA

There is strong evidence that synovitis is associated with further worsening of OA structure. Longitudinal analyses of 531 knee OA patients demonstrated that ultrasound-detected effusion predicted pain, radiographic progression and also joint replacement [45]. There are also cohorts reporting higher risk of radiographic progression and cartilage deterioration over 2 years in knee joints with increased MRI synovitis score over time than those with a decrease in synovitis [39, 46].

Studies of hand OA patients report similar associations. Using ultrasound, Mathiessen et al. [29] found grey-scale synovitis and power Doppler signals at baseline to strongly predict radiographic progression after 5 years, and even more so when inflammation persisted [47]. Similar results were found in two cohorts using MRI on hand OA patients, as gadolinium-enhanced synovitis was associated with both onset and progression of radiographic hand OA, including development of erosions [48, 49]. The total amount of synovial inflammation also increased the risk of progression [49].

## Treating OA synovitis—what have we learnt?

### The data presented strongly suggest that synovitis is involved in OA symptoms and progression, and therefore represents an important target for therapeutic intervention.

While symptoms are the usual primary outcome of an analgesic OA trial, imaging or serum biomarkers of synovial inflammation may provide evidence for proof of mechanism [15]. Synovial tissue volume was used for this purpose in a recent open-label study of 120 patients with knee OA receiving intra-articular (IA) steroid injection with subsequent reduction in CE-MRI synovial volume that correlated with improvement in knee pain [50]. Dynamic CE-MRI-derived measurements of synovial enhancement, where images are acquired every few seconds after contrast injection, were shown to be even more sensitive to the response of treatment and more strongly associated with changes in pain than synovial tissue volume [51, 52].

### Existing therapies

Part of the evidence to support therapeutic targeting of inflammation in OA is that the two pharmacological therapies with consistently good effect sizes in relieving OA pain are NSAIDs and IA corticosteroid injections [53]; both have anti-inflammatory mechanisms of action.

Further support for the concept of treating inflammation is that the analgesic benefits from IA steroids also seem dose dependent. In a hip OA study where 120 patients received either 40 or 80 mg of IA corticosteroids, both doses had a beneficial effect at week 6 (in terms of pain, stiffness and disability), while the 80 mg dose demonstrated a statistically significant benefit at week 12 [54]. This stands in contrast to low-dose oral prednisolone (5 mg) which had no analgesic effect on 70 hand OA patients in a short-term (4 weeks) randomized controlled trial [55]. Intramuscular depot corticosteroid demonstrated somewhat better results with significant short-term effects on knee pain [56], although no statistically significant reduction in ultrasound-detected synovial inflammation was found [56]. A preliminary report of a novel slow-release, microsphere formulation of triamcinolone in a large OA knee randomized controlled trial demonstrated important analgesic benefits to 13 weeks, offering hope of long-lasting IA steroid therapy [57].

### Potential therapies

Methotrexate (MTX) has an anti-inflammatory effect by suppressing the inflammatory functions of neutrophils, macrophages and monocytes, dendritic cells and lymphocytes through adenosine release, and thereby reducing secretion of inflammatory cytokines, including TNF-α and IL-6. A recent open-label pilot study of 30 patients with knee OA who took a MTX dose of 15–20 mg/week for 6 months suggested an analgesic benefit, with 43% achieving the Osteoarthritis Research Society International (OARSI) responder criteria [58]. A subsequent randomized controlled trial is underway.

A recent review has summarized the role of biologic therapies that modify inflammatory components, including anti-TNF agents, growth factors and IL-1 antagonists, and their reported effects in OA trials [59]. Anti-TNF agents have to date had minimal effects on symptoms, although the trial designs are heterogeneous and there may be a possible structural-modifying effect in joints with the most inflammation [60]. Also, there are emerging and promising data on anti-inflammatory cytokines such as IL-4 and IL-10, as well as anti-CSF therapies, as described earlier.

## Conclusions

Even allowing for discrepancies between studies, synovitis is a common finding in OA joints and has been associated with clinical symptoms; our current best analgesic therapies work by anti-inflammatory mechanisms supporting the benefits of targeting inflammation to reduce pain. Overall evidence suggests that synovial inflammation is associated with progressive joint failure at least in a subgroup of patients, and thus some OA patients may benefit structurally from an anti-inflammatory intervention. The failure to identify and select subgroups of patients who will benefit most may be the reason why treatment trials targeting inflammation have failed to show convincing and long-lasting effects on structure modification and symptoms, but advances in imaging and soluble biomarkers may well provide the tools for appropriate stratification and understanding the mechanism of action. More work is required on the molecular pathways initiating and perpetuating synovial inflammation, because dissection of these pathways will provide novel therapeutic opportunities.

## Abbreviations

ADAMTS: a disintegrin and metalloproteinase with thrombospondin motifs
AUSCAN: Australian/Canadian Osteoarthritis Hand Index
BML: bone marrow lesion
C1M: connective tissue type I collagen turnover
CE: contrast-enhanced
CI: confidence interval
CRPM: matrix metalloproteinase-dependent degradation of C-reactive protein
CSF: Colony-stimulating factor
IA: intra-articular
ICOAP: Intermittent and Constant Osteoarthritis Pain
IL: interleukin
KOOS: Knee injury and Osteoarthritis Outcome Score
MGC: Multinucleated giant cell
MMP: matrix metalloproteinase
MOST: Multicenter Osteoarthritis Study
MRI: Magnetic resonance imaging
MTX: Methotrexate
OA: Osteoarthritis
OAI: Osteoarthritis Initiative
OARSI: Osteoarthritis Research Society International
OR: odds ratio
SF-36: Short Form 36
TNF: tumour necrosis factor
VAS: visual analogue scale
WOMAC: Western Ontario and McMaster Universities Arthritis Index
